# Visualizing Nudivirus Assembly and Egress

**DOI:** 10.1128/mBio.01333-20

**Published:** 2020-08-11

**Authors:** Sailakshmi Velamoor, Allan Mitchell, Bruno M. Humbel, WonMo Kim, Charlotte Pushparajan, Gabriel Visnovsky, Laura N. Burga, Mihnea Bostina

**Affiliations:** a Department of Microbiology and Immunology, University of Otago, Dunedin, New Zealand; b Otago Micro and Nano Imaging, Electron Microscopy, University of Otago, Dunedin, New Zealand; c IMG, Okinawa Institute of Science and Technology, Okinawa, Japan; d Department of Chemical and Process Engineering Department, University of Canterbury, Christchurch, New Zealand; Columbia University Medical College

**Keywords:** electron microscopy, enveloped viruses, nudiviruses, viral assembly

## Abstract

Enveloped viruses hijack cellular membranes in order to provide the necessary material for virion assembly. In particular, viruses that replicate and assemble inside the nucleus have developed special approaches to modify the nuclear landscape for their advantage. We used electron microscopy to investigate cellular changes occurring during nudivirus infection and we characterized a unique mechanism for assembly, packaging, and transport of new virions across the nuclear membrane and through the cytoplasm. Our three-dimensional reconstructions describe the complex remodeling of the nuclear membrane necessary to release vesicle-associated viruses into the cytoplasm. This is the first report of nuclear morphological reconfigurations that occur during nudiviral infection.

## Abstract (precis)

The dynamics of nuclear envelope has a critical role in multiple cellular processes. However, little is known regarding the structural changes occurring inside the nucleus or at the inner and outer nuclear membranes. For viruses assembling inside the nucleus, remodeling of the intranuclear membrane plays an important role in the process of virion assembly. Here, we monitored the changes associated with viral infection in the case of nudiviruses. Our data revealed dramatic membrane remodeling inside the nuclear compartment during infection with *Oryctes rhinoceros nudivirus*, an important biocontrol agent against coconut rhinoceros beetle, a devastating pest for coconut and oil palm trees. Based on these findings, we propose a model for nudivirus assembly in which nuclear packaging occurs in fully enveloped virions.

## INTRODUCTION

Enveloped viruses hijack cellular membranes during viral replication, a process that becomes essential during virion assembly. This maneuver offers the viral genome both a straightforward mechanism for viral entry and the necessary protection in the environment or in the extracellular space of the host organism. The strategy is used by numerous viruses with various hosts, replication mechanisms, or types of genomic material. Double-stranded DNA (dsDNA) viruses represent half of the known virus species with an identified host (1); nevertheless, they are less diverse and less abundant in eukaryotic hosts. The abundance of membranous structures in cytoplasm makes this compartment the preferred site for viral replication for RNA viruses and some dsDNA viruses. The dsDNA viruses that replicate in the nucleus have evolved special mechanisms to modify the nuclear landscape during infection (1). For enveloped viruses in particular, sourcing the necessary membrane for the assembly of complete virions inside the nucleus poses additional barriers.

Large dsDNA rod-shaped enveloped viruses are a major group of invertebrate pathogens consisting of four families: *Baculoviridae*, *Nudiviridae*, *Hytrosaviridae*, and *Nimaviridae* (2–4). They all have large circular dsDNA genomes and share a set of essential genes suggesting a common evolutionary origin. Electron microscopy (EM) studies have shown that members of this group assemble inside cell nucleus (5–8). Little is known of how these viruses remodel the nuclear membrane in order to envelope the nucleocapsid. Most of the efforts were focused on understanding baculovirus assembly (8, 9) and have shown a combination of nuclear envelope reorganization and production of occlusion bodies destined to protect the virus in the environment.

Nudiviruses display a wider host range than baculo-, hytrosa-, and nimaviruses, infecting multiple insects and members of the crustacean orders (4, 10). Genomic analysis initially classified nudiviruses as baculoviruses. However, the observation that these viruses do not form occlusion bodies has imposed the revision of the taxonomy and placed them in an independent family, the prefix reflecting that the virions emerge naked from the infected cells. Nudiviruses have long shaped enveloped virions of various lengths containing a single nucleocapsid. The sizes of their genomes vary considerably between 97 and 232 kbp (4). A total of 32 genes were found to be common for all nudiviruses, 21 of which are also shared with baculoviruses (11). Nevertheless, the relation between the two families proved to be more complicated as baculoviruses can assemble into a well-characterized nonoccluded form (12), while some nudiviruses could also form occlusion bodies during replication (11).

The best characterized member of *Nudiviridae* is *Oryctes rhinoceros nudivirus* (OrNV) (13). OrNV has a 128-kbp genome encoding 139 open reading frames (14). The enveloped, rod-shaped virus was first observed in the nuclei of fat body cells of infected larvae of the coconut rhinoceros beetle *Oryctes rhinoceros* (13). The virus is orally transmitted by infection of larvae and adult. The infection starts in midgut epithelial cells, from where it spreads to other tissues (15). The fact that beetle population is drastically reduced after infection made OrNV a widely used biocontrol agent, which helps the recovery of badly damaged coconut and oil palm trees in Southeast Asia and the Pacific Islands (16). Establishment of DSIR-HA-1179, a susceptible and permissive host cell line that was isolated from the black beetle *Heteronychus arator*, has proven to be an important step toward producing a higher yield of viable OrNV (17–19). Although the timeline for virus entry, replication, and budding has been previously described (5), the mechanism behind viral replication and assembly is still unclear. In this study, we used several EM techniques to describe the cellular changes during OrNV infection and the process of viral assembly and to characterize the membranous architectures induced by the virus in the nucleus during nucleocytoplasmic transport and viral egress.

## RESULTS

Despite the genomic similarities between baculoviruses and nudiviruses, the differences in their replication strategies are significant (4, 12). The large arsenal of genetic manipulation tools available for different species belonging to the *Baculoviridae* family has allowed to decipher the role of specific genes at diverse stages of their life cycle (10). In the case of nudiviruses where such a system is not yet available, electron microscopy is a powerful tool that can be used to uncover the mechanism of viral assembly. We used high-pressure freezing and freeze substitution (HPF-FS), followed by electron microscopy, volume microscopy, and cryo-electron microscopy (cryo-EM), in order to offer a detailed description of the ultrastructural changes that occur during nudivirus replication.

### Cellular morphology during infection.

**FIG 1:**
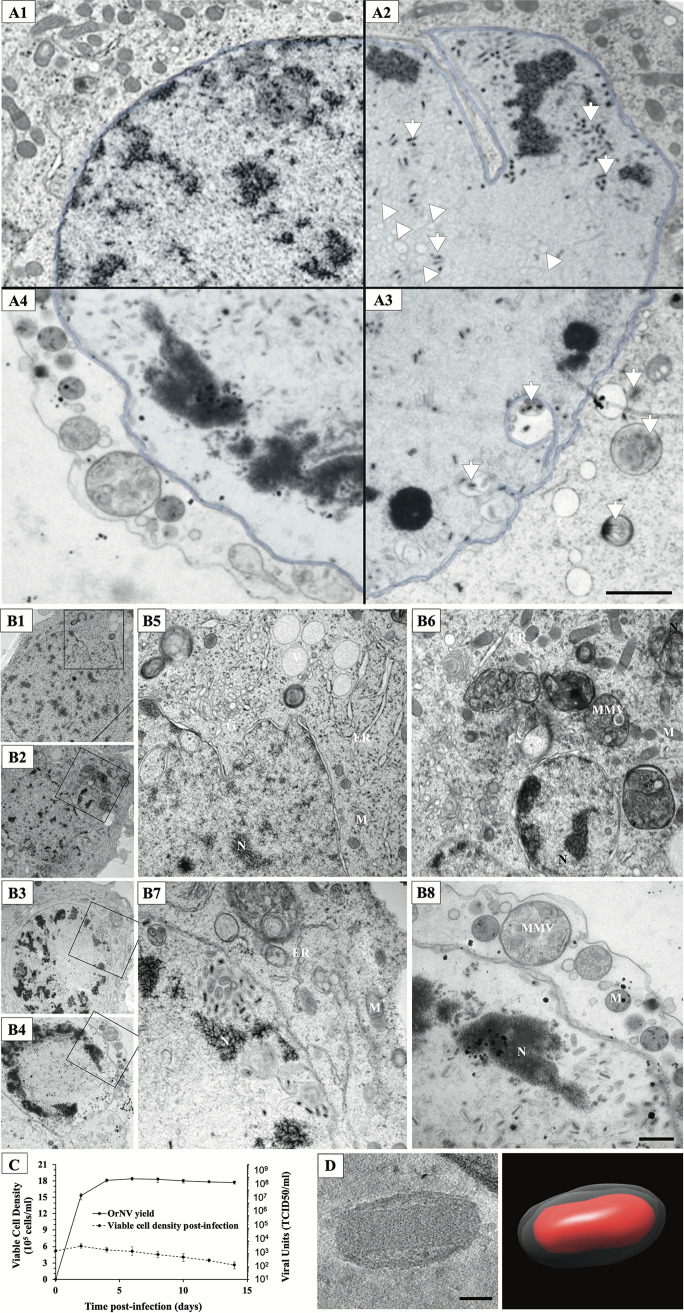
Infection of insect cells with OrNV. (A) Stages of infection. (A1) Uninfected cell with intact nucleus. (A2) At midinfection, few virions (white arrows) surrounded by newly evolved vesicles (arrowheads) are observed inside the nucleus. (A3) In late infection the nuclear membrane which appears slightly distorted undergoes major changes to assist the assembly and the transport of virions. (A4) Cell lysis occurs around 72 h postinfection (hpi). Scale bar, 1,000 nm. (B) Comparison of nucleus and cytoplasmic organelles in OrNV-infected cells to uninfected cells. (B1) Low magnification of uninfected HA cell. High magnification of the inset (B5) shows that mitochondria, Golgi bodies, and ER remain intact in uninfected HA cells. (B2) HA cells infected with (low dose) OrNV at 16 hpi. While normal Golgi and ER are seen at a higher magnification (B6), the mitochondrial structures appear to be affected. Large MMV are also observed in the cytoplasm when virion replication and budding from plasma membrane peaked. (B3) OrNV-infected cells at 72 hpi (high dose). At a higher magnification (B7), the cytoplasm seems to be less electron dense, and organelles such as Golgi bodies and the ER seem to be disrupted. (B4) Infected HA cells resembling the late infection phase. Inset, at a high magnification (B8), shows no evident intact Golgi or ER structures. The scale bar of low-magnification micrographs (B1 to B4) represents 1,000 nm. The scale bar of images from inset (B5 to B8) represents 500 nm. (C) Time course of OrNV infection showing the evolution of viral titers and the concomitant decrease in viable cell density postinfection. (D) Cryo-EM of OrNV shed into the extracellular space showing the capsid surrounded by a thick envelope and its segmentation (right panel). Scale bar, 50 nm.

Cell cultures were nonsynchronously infected with OrNV; therefore, infection was allowed to progress for 72 h for the majority of cells in culture to become infected in secondary infection before the culture was sampled to obtain a complete overview of OrNV replication steps. Based on visual inspection of electron microscopy images of over a hundred well-preserved HPF-FS cells and comparing cellular changes from time point experiments (data not presented) with previous detailed ultrastructural studies (5, 15), we identified different stages of infection characterized by clear morphological features (Fig. 1A). Uninfected DSIR-HA-1179 cells showed a round appearance about 5 μm in diameter with elongated mitochondria and a nucleus with a smooth nuclear membrane (Fig. 1A, B1, and B5). As infection progressed, the nucleus become filled with virions. The Golgi compartment and endoplasmic reticulum (ER) lost their integrity, giving rise to collapsed structures and numerous vesicles (Fig. 1B2 to B4, B6, and B8). The vesicles evolved gradually (Fig. 1A2) and became larger, more numerous, and enclosing virions in the later stages (Fig. 1A3). The mitochondria became rounder and less abundant. Finally, the cells completely lost their integrity (Fig. 1A4, B4, and B8), releasing nuclear and cytoplasmic material containing virions into the extracellular space.

Strategies to overcome slow growth kinetics of the DSIR-HA-1179 cell line and inconsistencies in productivity using traditional approaches of virus production were previously reported (5, 20, 21). By monitoring of virus titer in insect cells infected with OrNV at a multiplicity of infection of five, we observed an increase of virus production up to day six postinoculation. Concurrently, the viable cell density in infected cultures steadily decreased from day 2 postinoculation (Fig. 1C). However, approximately half of the initial number of cells were still viable at 2 weeks postinfection. In our EM imaging experiments, we used a high multiplicity of infection of OrNV to ensure a majority of cells were infected, and the virions were easily visible in the EM sections.

### Characterization of virions.

In order to observe the OrNV particles expelled into the extracellular space, virions were purified from the cell culture media. The purification was confirmed by negative staining and analyzed by cryo-ET (Fig. 1D). The tomograms showed ovoid particles about ∼218 nm in length, with a maximum width of about ∼116 nm. The density of the material contained inside the viral envelope appeared darker, suggesting the presence of matrix proteins. The outline of the nucleocapsid was clearly distinguishable, with a constant width of ∼60 nm and a length of 200 nm. The capsid has a cylindrical silhouette with abrupt ends indicating distinct strategies to generate an elongated tubular structure and to cap the extremities. The size of the virions as measured in cryo-ET (Fig. 1D) is consistent with those from negative-stain and HPF-FS processed samples.

### Changes in the nuclear morphology.

**FIG 2:**
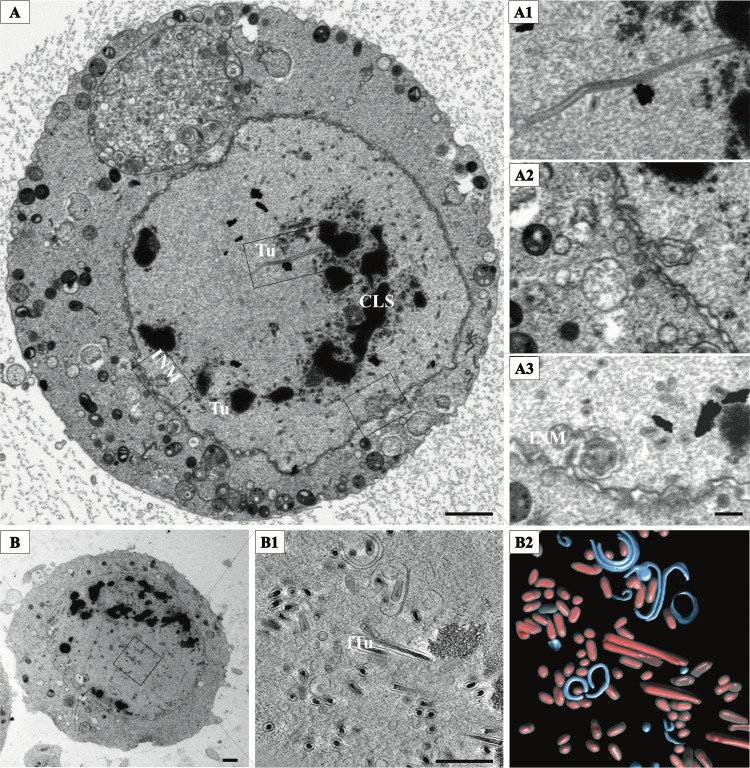
Intranuclear assembly of OrNV virions. (A) A slice from FIB-SEM reconstruction of an infected cell. Scale bar, 2 μm. (A1) The nucleus is filled with long tubules (Tu) extending from chromatin-like structures (CLS). Clusters of new virions associated with Tu migrate toward the inner nuclear membrane (INM). The INM invaginates inside the nucleus (A2), engulfing fully assembled virions (A3). Scale bar, 500 nm. (B) TEM micrograph of an OrNV-infected cell displaying the mechanism of virion assembly and virion enwrapping in vesicles (scale bar, 1 μm). Tomographic reconstruction of the region shown in the inset. (B1) A 11-nm-thick section showing long filled tubules (fTu) and virion clusters being enveloped in membranes. Scale bar, 500 nm. (B2) Segmentation of the tomogram displaying viral capsids (red) inside filled tubules (gray) and membrane structures (blue).

Volume electron microscopy and electron tomography (ET) were employed to gather more details on changes of the nuclear structure in areas of special interest. We observed that postinfection, the chromatin starts to segregate into electron dense zones with granular domains of 1 to 2 μm in diameter, and virogenic stroma (VS) characterized by less-electron-dense areas associated with viral replication. The nucleus becomes populated with tubular membrane structures and clusters of virions around them (Fig. 2A). These membrane tubules can reach several micrometers in length and appear to be scattered throughout the volume of nucleus (Fig. 2A1; see also Fig. S1 and Video S1 in the supplemental material).

10.1128/mBio.01333-20.3 FIG S1 Volume electron microscopy displaying long tubules scattered around the entire volume of the nucleus at 72 hpi. (A) Micrographs display tubules, approximately 2 to 3 μm long, near the nuclear membrane (A and B) and at the center of the nucleus (C). Insets are 36-nm-thick tomographic slices at higher magnification. Only part of the tubule is seen in image B, while the inset shows a full length of tubule, as the volume in the inset is rotated (by 3.6° and 0.1°) along *x* and *z* axis. Scale bar for images A, B, and C (and their insets), 1,000 nm. (D) Segmentation of the volume, tilted at 45°, displays tubules scattered around the nucleus. Scale bar, 2,000 nm. Download FIG S1, PDF file, 0.7 MB. Copyright © 2020 Velamoor et al. 2020 Velamoor et al. This content is distributed under the terms of the Creative Commons Attribution 4.0 International license.

10.1128/mBio.01333-20.1 VIDEO S1 Volume electron microscopy of OrNV-infected cells to uninfected cells. Enveloped virions replicating inside the nucleus are engulfed by the INM, transported into the NE before being transported into the cytoplasm. Inside the cytoplasm, virions either appear alone or inside multimembrane vesicles. Eventually, they all escape into the extracellular environment. Scale bar, 2,000 nm. Download Movie S1, MOV file, 15.4 MB. Copyright © 2020 Velamoor et al. 2020 Velamoor et al. This content is distributed under the terms of the Creative Commons Attribution 4.0 International license.

**FIG 3:**
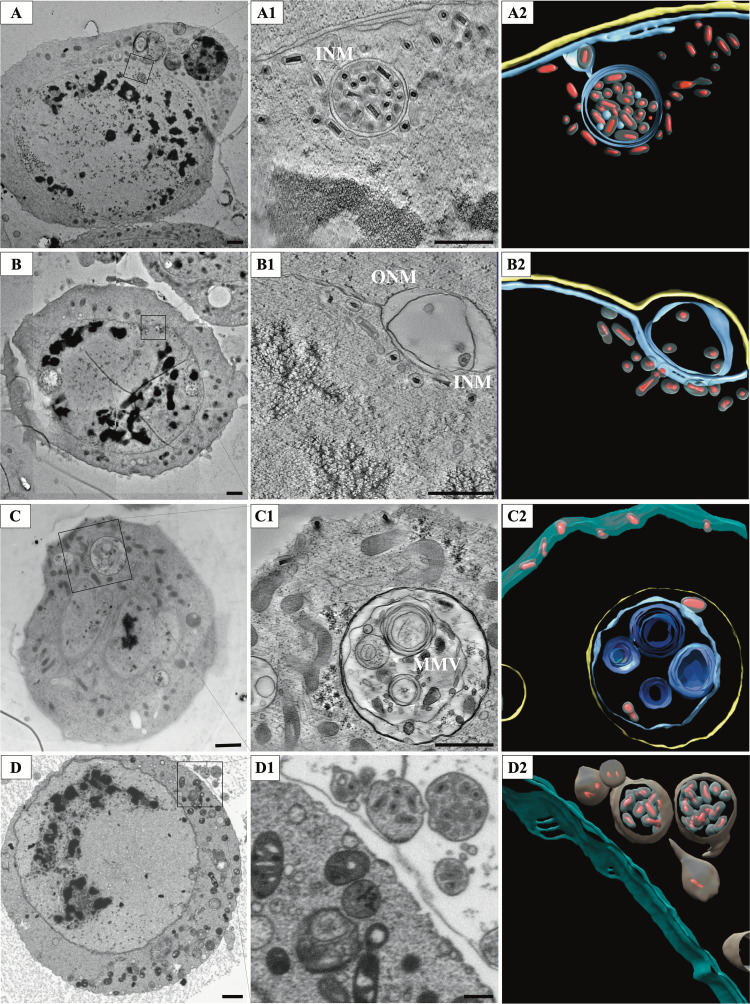
Virion trafficking inside the cell. (A) Micrograph of an OrNV-infected cell showing virions encapsulated into a vesicle inside the nucleus. Scale bar, 1 μm. (A1) A 11-nm slice through the tomographic reconstruction indicated in inset showing virions enclosed in a double membrane vesicle. Scale bar, 500 nm. (A2) Segmentation of the tomogram. (B) Micrograph displaying expanded NE. Scale bar, 1 μm. (B1) A 11-nm slice through the tomographic reconstruction indicated in inset shows virions inside an expanded NE lumen. Scale bar, 500 nm. (B2) Segmentation of B1 highlights vesicle wrapped virions inside the NE lumen. (C) Micrograph shows cytoplasm completely traversing the nucleus. Scale bar, 1 μm. (C1) A 11-nm slice through the tomographic reconstruction of the area indicated in inset displaying virions encapsulated into multimembrane vesicles (MMVs) in the cytoplasm. Scale bar, 500 nm. (C2) Segmentation showing virions inside complex MMVs, and vesicle-free virions close to the cellular membrane. (D) An infected cell showing vesicles being expelled into the extracellular space. Scale bar, 1 μm. (D1) A slice through the tomographic reconstruction of the area indicated in inset showing vesicles containing fully enveloped virions. Scale bar, 500 nm. (D2) Segmentation showing virions within vesicles.

Large vesicles, both with and without viruses, appear inside the nucleus close to the nuclear membrane (Fig. 2A2). Electron tomography and volume electron microscopy revealed protrusion of both the inner nuclear membrane (INM) and the outer nuclear membrane (ONM) inside the nucleus (Fig. 2A2 and see Fig. S2 in the supplemental material). These finger-like invaginations, resembling the type II nucleoplasmic reticulum (22), brought the cytoplasmic material in close proximity to nucleus center. This geometry was shown to allow local delivery of cytoplasmic proteins through the nuclear pore and is common in the advanced stages of infection. We observed that these finger-like protrusions were often connected to small vesicles with virions, suggesting that they could engulf nuclear material. Some of these invaginations completely traversed the nucleus (Fig. 3C; see also Video S1 in the supplemental material). Also, due to their angle respective to the sectioning plane, they could appear as double-membraned vesicles (Fig. S2). In the last stage of infection, the nuclear membrane becomes distorted and the original nuclear structure is being gradually lost (Fig. 1A4).

10.1128/mBio.01333-20.4 FIG S2 Invagination of the cytoplasm into the nucleus. (A) Cross-section of a cell showing cytoplasmic components invaginating into the nucleus. (B) Cytoplasmic organelles, such as ribosomes, can be clearly seen inside these invaginations at a higher magnification in the inset. Due to level of section cut, these invaginations (*) from cytoplasm (Cy), sometime encapsulating the virions (indicated by arrows), appear as double membrane vesicles. Numerous microvesicles noticed inside the nucleus are indicated arrowheads. Scale bar, 1,000 nm. Download FIG S2, PDF file, 0.3 MB. Copyright © 2020 Velamoor et al. 2020 Velamoor et al. This content is distributed under the terms of the Creative Commons Attribution 4.0 International license.

### Virion assembly.

Viral assembly and shedding is observed long time before the actual cell death, indicating that most of cellular processes continue simultaneously with virus production (Fig. 1C). In line with this observation, we saw that cells with no obvious cytopathic effect were harboring virus particles inside the nucleus, dispersed around chromatin aggregates or in the cytoplasm, either alone or inside vesicles (Fig. S3). However, the presence of a large number of virions was associated with dramatic changes in the nuclear morphology, indicating that ample and efficient virion assembly requires a significant remodeling of the membrane environment (Fig. 2). Enveloped virions did not seem to be confined to a specific domain but were found disseminated through the whole nuclear space. Nevertheless, defined areas with a high density of fully formed virions occur frequently and are associated with an abundance of membrane material. Our ET reconstructions indicate clustering of numerous microvesicles in regions adjacent to enveloped virions (Fig. 2B1 and Fig. S2). This mechanism is similar to that used by baculoviruses where the microvesicles supply the necessary membrane material for enveloping the viral nucleocapsids (9). Differently from baculoviruses, where several nucleocapsids could be contained in a single envelope (8, 23), each OrNV virion contains a single capsid, making the demand for membrane material much higher. This is provided by the numerous INM or nuclear envelope (NE) invaginations that are capable to deliver membrane material toward the central part of the nucleus where assembly takes place (Fig. 2B2).

10.1128/mBio.01333-20.5 FIG S3 Distribution of virions in interchromatin space and cytoplasm. A micrograph of a cell showing virions dispersed in the nucleus and cytoplasm is shown. The inset at a higher magnification clearly shows that cytoplasmic organelles are not affected during virions replication and assembly. Virions appear scattered throughout the nucleus (N) and cytoplasm (Cy) either alone (arrowhead) or inside vesicles (arrows). Scale bar, 1,000 nm. Download FIG S3, PDF file, 2.1 MB. Copyright © 2020 Velamoor et al. 2020 Velamoor et al. This content is distributed under the terms of the Creative Commons Attribution 4.0 International license.

Another particularity of nudivirus assembly sites is the frequent presence of continuous long membrane tubules. A careful inspection of our ET reconstructions reveals a dense layer inside each tube, suggesting that the tubules are modeled into a stable geometry by an internal protein layer that ensures a diameter consistent with virion dimensions both for the viral envelope and the nucleocapsid (Fig. 2B1 and 2B2). This design leads us to propose that the membrane tubules are used for subsequent DNA packaging. The extremely rare cases of nonenveloped and filled nucleocapsids observed either inside the nucleus or in the cytoplasm of infected cells at all stages of infection argue for the idea that nucleocapsids are enveloped simultaneously with the genome packaging. This scenario would require a series of proteins that regulate the assembly process and decide either to “engage” and start DNA packaging or to “cap” and release fully formed virions. Indeed, the fact that membrane tubules can be commonly observed having a dense core with similar density and dimensions as the full virions supports this hypothesis (Fig. 2B1). These filled tubules could be defective DNA packaged virions for which the regulatory process went wrong. Alternatively, the releasing of fully formed virions could occur at one end of the tubule, while the packaging could continuously provide material at the other end. Consistent with this alternative is the fact that we have occasionally observed fully formed virions being released from one end of a full tubule (see Video S2 in the supplemental material). The proposed mechanism implies that genome encapsidation is simultaneous with both capsid formation and enveloping. Therefore, both virion assembly and maturation must take place in the nuclear compartment, since in no instance were these tubules observed inside nuclear vesicles or in the cytoplasm. While this hypothesis has still to be tested, it implies that OrNV is using an approach totally different from related viruses, where recruitment of the viral membrane is done after the genome packaging was completed.

10.1128/mBio.01333-20.2 VIDEO S2 Budding of virions from tubules. A tomogram of OrNV-infected cell releasing virions (indicated by arrows) from long tubules inside the nucleus (N) is shown. Scale bar, 500 nm. Download Movie S2, MOV file, 5.3 MB. Copyright © 2020 Velamoor et al. 2020 Velamoor et al. This content is distributed under the terms of the Creative Commons Attribution 4.0 International license.

### Nucleocytoplasmic transport and viral egress.

Continuous shedding of infectious virions before cell lysis requires an efficient trafficking mechanism from the nucleus through the NE and cytoplasm and the final shedding from the plasma membrane. The abundance of membrane structures associated with OrNV infection facilitates this transport. Numerous vesicles, some containing virions, were visible from the early stages of infection. Their number, size, degree of complexity and virion load increased as the infection progressed. Intranuclear protrusions of the nuclear membrane were always associated with an abundance of viral particles, including large vesicles containing a variable number of fully assembled viruses. These vesicles have a diverse topology ranging from single membrane to complex multimembrane vesicles.

The nucleocytoplasmic transport of fully enveloped OrNV virions poses a series of specific problems distinct from other DNA viruses replicating inside the nucleus. For baculoviruses and herpesviruses, the interaction between capsids proteins and membranes or associated membrane proteins are speculated to govern the envelopment of the virion and to mediate the exit from the nucleus or the cell (10, 24). However, in the nudivirus case, these processes are coordinated by the viral envelope proteins. The nuclear exit involves the task of passing a double membrane while retaining the viral envelope. Engulfment of virions by finger-like structures derived from INM allows the access to the intermembrane compartment. We have observed numerous instances of virions encapsulated in vesicles connected to NE lumen (Fig. 3A). The load of these vesicles will find its way toward the ONM as the continuous demand of membrane for virion assembly will stretch the NE simultaneously with the expansion of the nuclear space (Fig. 3B). The release of the large vesicles at ONM will place virions either directly in the cytoplasmic space or transfer them inside a multimembrane vesicle (Fig. 3C; see also Fig. S4 in the supplemental material). At the resolution attained using our imaging techniques, the transport does not seem to alter the virion morphology.

10.1128/mBio.01333-20.6 FIG S4 Exvagination of virions into the cytoplasm. (A) Electron micrograph displaying virions expelled into the cytoplasmic region (indicated by arrow). Association of outer nuclear membrane (ONM) with the multi membrane vesicles (MMV) is indicated with an arrowhead. (B) A 30 nm tomographic slice shows that every single virion, inside the nuclear envelope (NE), is enclosed within a vesicle, exvaginating into a MMVs. Scale bar, 500 nm. Download FIG S4, PDF file, 1.0 MB. Copyright © 2020 Velamoor et al. 2020 Velamoor et al. This content is distributed under the terms of the Creative Commons Attribution 4.0 International license.

Nuclear exit was common in most of the inspected cells regardless of the stage of infection. Numerous vesicle-free virions were observed to exit the plasma membrane individually (Fig. 3C; see also Video S1). Equally, multiple membrane vesicles (MMVs) were frequently budding out into the extracellular space in an exosome-like fashion, releasing virions enclosed in one or more membrane layers (Fig. 3D).

Cell lysis follows OrNV infection after 72 h, as proven by our growth curves and the numerous lysed cells observed in our experiment (Fig. 1A4). At this stage, the nucleus appeared distorted and filled with mostly empty vesicles. Despite these distortions, long tubules were still present inside the nucleus. The cytoplasm was completely damaged, and few single free-floating virions were observed. As the plasma membrane was ruptured, all the dead organelles along with the virions, either individually or inside MMVs, escaped easily into the extracellular space (see Fig. S5 in the supplemental material).

10.1128/mBio.01333-20.7 FIG S5 Cell lysis at 72 hpi with OrNV. (A) Electron micrograph of an OrNV-infected cell undergoing lysis. (B) High magnification of the inset showing individual free-floating virions in the distorted nucleus (N), indicated with an arrow. The cellular membrane is also ruptured; however, MMVs with few virions are still present. Scale bar, 1,000 nm. Download FIG S5, PDF file, 1.1 MB. Copyright © 2020 Velamoor et al. 2020 Velamoor et al. This content is distributed under the terms of the Creative Commons Attribution 4.0 International license.

## DISCUSSION

The strategies required by OrNV to replicate, assemble and egress pose a series of specific challenges. Some aspects of nuclear remodeling during OrNV replication resemble changes observed in baculovirus infections, with whom it shares a subset of the 38 core genes (8, 10). For instance, the formation and general appearance of VS, a region in the nucleus from which host chromatin is excluded, resembles the behavior observed in cells infected by baculoviruses (25). In late stages of infection, VS occupies most of the nuclear space, while the chromatin is condensed in small islands sparsely distributed. Numerous virions are spread across the entire nucleus, and densely packed regions that could reach several microns in length are common in later stages of infection.

**FIG 4:**
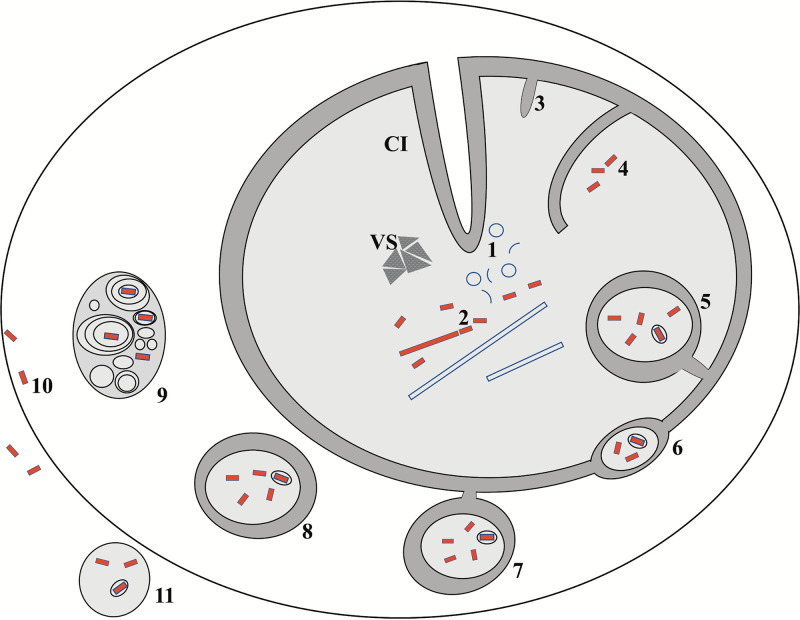
Proposed model for nudivirus assembly and trafficking. (Step 1) Membrane material is accumulated at the replication sites as small vesicles and membrane fragments. Cytoplasmic invaginations (CI) facilitate membrane presence deep inside nucleus. (Step 2) Virion assembling sites occur adjacent to virogenic stroma (VS) and are populated with Tu and fTu, from which the new virions are seen budding out. Inner nuclear membrane (INM) forms finger-like structures (step 3) into the nucleus and growing larger (step 4), eventually engulfing fully formed virions (step 5). (Step 6) Virion filled vesicles are transported into the NE lumen. Budding at the outer nuclear membrane (ONM) (step 7) releases multimembrane vesicles inside the cytoplasm (step 8). (Step 9) Inside the cytoplasm virions can be encapsulated into multivesicular bodies released into the extracellular space in an exosome-like fashion. (Step 10) Membrane fusion in this compartment can occasionally release virions inside the cytoplasm. (Step 11) Fusion on the plasma membrane can release virion filled vesicles into the extracellular space.

Other aspects of nuclear remodeling point toward a unique strategy for OrNV assembly. The presence of long membrane tubules is a special case. At first sight, they remind of the assembly factories reported in the nuclei of polyomavirus-infected cells (26), where tubular structures are adjacent to clusters of assembled virions. Using ET, it was shown that polyoma virions apparently are “budding” from the ends of the tubules. Despite these similarities, the two mechanisms have important differences. For polyomavirus, an icosahedral nonenveloped virus, the tubular structure is formed by major capsid proteins that change their organization from tubular to icosahedral in the process of genome-packaging (26). In OrNV, the tubular structure involves both membranes and proteins in which the tubules may provide the final material ready to be used for genome packaging (Fig. 4). The tubular diameter is consistent with that of the mature virions, and the presence of tubules in areas rich with fully enveloped virions supports this hypothesis. This strategy would involve a complex mechanism that simultaneously combines DNA packaging with membrane remodeling and capsid maturation. Many of the pieces required by such a mechanism are already present in baculoviruses. It was shown that baculovirus genome packaging occurs in the presence of membranes where already capped capsids align perpendicular to large vesicles in order to receive the dsDNA (9). The angular ends of OrNV capsid shown by our cryo-ET is similar to the basal side of baculovirus virions (23), suggesting that specific molecular complexes are responsible for “closing” the tubes. Little is known regarding the baculovirus proteins responsible for building the apical and basal complexes (27). There is ample data showing that deletion of different genes involved in baculovirus assembly can induce the growth of long tubules (27–29), and expression of baculovirus capsid protein generates long protein tubes with helical symmetry (30). Even more, in the absence of genomic material, the presence of the capsid protein alone is sufficient for the formation of helical tubular structures (27). Of particular interest in the case of OrNV is the involvement of nuclear membranes in this process (Fig. 4). In contrast to baculoviruses, our data show that long OrNV protein tubules always have a membrane envelope. Due to the genetic similarity between baculoviruses and nudiviruses it is expected that the OrNV capsid proteins have the same capacity to polymerize in helical assemblies. At the same time, they must be able to recruit membrane material, either alone or via some yet to be identified matrix proteins.

Of major interest for the study of rod-shaped viruses is the regulation of their capsid length. Does the size of the genome dictate the dimension of capsids, or are the capsids formed with a fixed morphology and subsequently filled with dsDNA? The existence of empty baculovirus capsids of a fixed length indicates a regulatory mechanism of capsid size. However, our observation of elongated filled tubules in the nuclei of OrNV-infected cells seems to favor the scenario of an assembly strategy capable to accommodate different genomic sizes. It is worth mentioning that rod-shaped dsDNA viruses have a wide range of lengths. Baculovirus could vary between 230 and 385 nm (31), the nudivirus HZ-1 is 371 nm (32), and hytrosaviruses can reach up to 600 nm in length (3). This dimensional variability supports the existence of a general mechanism capable to adapt to packaging of diverse sizes of viral genomes.

Apart from the lack of occlusion bodies, the mechanism of shedding mature virions is another particularity of OrNV compared to baculoviruses. The release of infectious virions before cell lysis is a common theme for dsDNA enveloped viruses replicating inside the host nucleus. Such a strategy is common for nudiviruses, baculoviruses, and herpesviruses (5, 24, 33, 34). For this, the infected cell undergoes a great extent of nuclear reorganization in order to accommodate fusion of virions with the INM and to transport them inside the NE lumen (24, 34–37). Invagination of either the INM alone (type I nucleoplasmic reticulum [NR]) or both INM and ONM (type II NR) can occur in normal cells and is abundant during virus infection. The cytoplasmic core of type II NR was shown to contain vesicles and MMVs, along with other cytoplasmic organelles such as ribosomes (38). This geometry able to bring cytoplasmic components closer to the interior of the nucleus and could play a role in nucleocytoplasmic transport. Previous studies have identified both type I and II NR in the nuclei of herpesvirus-infected cells and were suggested to play a role in fusion of the virions with the INM (22). Electron micrographs and tomograms from the present study reveal both type I and II NR, suggesting that OrNV might use a similar mechanism to fuse the virions into the INM. Studies show that these invaginations are common in normal cells and are speculated to shorten the intranuclear diffusion (39). Although invaginations which traverse the nucleus and create tunnels were found associated with cytoskeletal filaments (40), we have not noticed any sign of such structures in our images. Inside the NE lumen the virions remain enveloped and enclosed inside vesicles. From the NE lumen, the vesicles containing virions bud-off through the ONM (Fig. 4). Tomograms also revealed that when in the cytoplasm, the vesicles show association with the endoplasmic reticulum (see Fig. S5). This is not surprising since the ER membrane is continuous with the ONM and the NE lumen is an extension of the ER lumen and access to ER-specific proteins will facilitate further viral trafficking. It is common for large DNA viruses to egress the nucleus through the ONM and/or ER (41, 42). Thus, it could be speculated that the OrNV is using both pathways to bud-off into the cytosol. In the cytoplasm, in addition to virions encapsulated in MMV, we have also noticed few vesicle-free virions, consistent with results reported previously (5).

The fact that OrNV is trafficked without losing its envelope during the nucleocytoplasmic transport raises some interesting questions. For viruses transiting the cytosol, such as herpesviruses or baculoviruses, the maturation takes place while acquiring cytosolic membranes or plasma membranes together with the necessary membrane proteins (34, 43). In the case of OrNV, there is no indication of the virion undergoing maturation in the cytoplasm before shedding into the extracellular space. Different from previous studies of other dsDNA viruses is the encapsulation of OrNV virions in cytoplasmic MMVs, a feature associated with RNA viruses (44, 45). Although the exact mechanism by which the nucleocapsids enter the cytoplasm is still unclear, baculoviruses were observed to acquire a double membrane vesicle derived from the nuclear membrane (36, 37, 43), but there are no reports of association with MMVs. However, in the present study, we observed the OrNV virions budding out of the ONM encapsulated in MMVs (Fig. 4). MMVs have been observed to be associated with both NE and NR, but their relationship is not clearly established (22, 46, 47). Endosomal sorting complex required for transport is reported to be responsible for biogenesis of MMV or multivesicular bodies that facilitate the budding and release of viruses from the host cell in an exosome-like fashion (44, 48). Viruses make extensive use of this mechanism to exit the cell (49). Considering that only uncoated nucleocapsids enter into the nucleoplasm for replication (5, 19), it could be speculated that the formation of MMVs and their association with both NR and NE might be crucial for OrNV transport into the cytoplasm. Importantly, the shedding of both free-floating and vesicle-enclosed virions from infected cells (Fig. 4) would offer the virus the advantage to escape the recognition by the host immune system and at the same time to be equipped to infect further cells via different entry mechanisms.

Our observations support the existence of a unique mechanism of viral assembly and egress for nudiviruses. Unlike other enveloped dsDNA viruses, OrNV acquires its envelope prior to packaging and maintains its envelope during trafficking across the cytoplasm and plasma membrane. OrNV uses both lytic and nonlytic pathways to release virions either as single units or contained in vesicles. This strategy is advancing the possibility to infect cells via two complementary approaches in a receptor-mediated manner or using a vesicle fusion mechanism. Our results highlight some important directions for research to better understand the nudivirus viral cycle. Adding genomic and proteomic data to our observation would help to unlock the exact mechanisms of viral entry, replication, assembly, and egress.

## MATERIALS AND METHODS

### Cell line and virus.

DSIR-HA-1179 cells were maintained at 27°C as attached cultures in 25-cm^2^ tissue culture flasks in TC-100 insect cell medium (Sigma) supplemented with 10% fetal bovine serum (FBS; Life Technologies, NZ). For long-term storage, 1 × 10^7^ DSIR-HA-1179 cells were resuspended in freezing medium (TC-100, 10% FBS, 10% dimethyl sulfoxide), allowed to freeze gradually using a Mr. Frosty freezing container (Nalgene) and later transferred to liquid nitrogen. Confluent monolayers of attached DSIR-HA-1179 cells at day 10 of culture were treated with TrypLE Express as previously described by Pushparajan et al. (19) to dissociate the cell monolayer into a single cell suspension. The cell suspension was counted on a hemocytometer. Approximately 2 × 10^5^ viable cells were transferred per well in a six-well plate (day 0) in a culture volume of 2 ml, followed by incubation for 5 days. At day 5, the cells in one well were dissociated with TrypLE Express and counted. Medium was replaced, and cells were inoculated with OrNV virus (strain X2B) at a multiplicity of infection of 2,000 virus particles per cell, followed by incubation at 27°C. After 72 h, the insect cells were detached, briefly centrifuged, resuspended in approximately 20 μl of growth medium, and subjected to HPF. Uninfected control cells were processed in the same manner.

*Oryctes nudivirus* strain X2B was propagated in DSIR-HA-1179 cells. Briefly, DSIR-HA-1179 cells grown to confluence in a T75 flask of were inoculated with OrNV virus stock and monitored for cytopathic effect. When the majority of cells presented a cytopathic effect, the flask with cells and medium was frozen (–80°C) and thawed at room temperature to release the virus from cells. The lysate was centrifuged for 5 min at 2,500 × *g*. Clarified lysate was quantified for OrNV infectious particles using the endpoint dilution method, aliquoted, and stored at –20°C.

For cryo-EM imaging of OrNV, 5 ml of cell medium of infected cells with cytopathic effect was filtered through a 40-μm cell strainer. The filtrate was incubated with 0.01% Triton X-100 for 2 min at room temperature to lyse the vesicles and concentrated by centrifugation at 3,000 × *g* at 4°C for 1 h through a 25-kDa filter (Millipore) prepared according to the manufacturer’s instructions. The virus in the concentrate (∼1 ml) was pelleted down by centrifugation at 21,000 × *g* at 4°C for 1 h, and the pellet was resuspended in 20 μl of PBS and used for EM imaging.

### High-pressure freezing.

Approximately 0.5 μl of concentrated cells were loaded into 200-μm-deep copper membrane carriers (Engineering Office M Wohlwend GmbH, Switzerland) precoated with hexadecane (Sigma-Aldrich). The membrane carriers with the sample were then transferred into the Leica EMPACT 2 Bayonet pod. Filler solution (10% Ficoll in 0.1 M cacodylate buffer) was added, and the transfer pod was torqued down and sealed. The samples were frozen using the EMPACT2 high-pressure freezing machine (Leica Microsystems, Vienna, Austria). The membrane carriers with frozen samples were removed from the transfer pod under liquid nitrogen and processed for freeze substitution.

### Freeze substitution.

For ultrastructural studies, the membrane carriers with frozen sample were transferred into a freeze-substitution apparatus (AFS2; Leica Microsystems) under liquid nitrogen and placed inside precooled sample molds. The molds were then filled with freeze substitution solution comprising 2% osmium tetroxide, 1% anhydrous glutaraldehyde, and 10% water in acetone precooled to –90°C. We found that a high water content improves the contrast membranes, as reported previously (50). The samples were incubated for 48 h. The temperature was then increased to –20°C over 7 h with a temperature slope of 10°C per h. The samples were held at –20°C for 12 h, and then the chamber temperature was slowly increased to 0°C over a period of 2 h and kept at 0°C for 30 min. Next, the samples were washed three times with acetone at 0°C, removed from the AFS2 into room temperature, and gradually infiltrated with increasing concentrations of epoxy resin (EMS EmBed 812) and then embedded in Epoxy EmBed 812 resin according to the manufacturer’s instructions.

For volume electron microscopy, samples were treated with various contrast-enhancing agents, as follows: after the acetone washes and removing the samples from the AFS machine, they were treated with 1% thiocarbohydrazine in acetone at 40°C (Acros Organics, 207530050) for 30 min, acetone washes at room temperature, and 2% OsO_4_ in acetone at 40°C for 30 and 60 min, respectively. After further acetone washes at room temperature, the samples were incubated with 2% uranyl acetate (in acetone) at 4°C overnight. The sample was than washed twice with acetone and twice with methanol at room temperature, followed by 2% lead acetate in methanol (Sigma-Aldrich, 215902) for an hour at 40°C. This last contrasting step was followed by methanol washes, methanol-acetone washes, and then acetone washes at room temperature. The samples were then gradually infiltrated with increasing concentrations of epoxy resin in acetone and finally embedded in Epoxy EmBed 812 resin according to the manufacturer’s instructions. The protocol following regular freeze substitution was kindly provided by Rick Webb (unpublished data) based on previously described methods (51).

### Ultramicrotomy.

The polymerized resin was sectioned using ultramicrotome (Leica UC6). Ultrathin sections (∼85 nm) were collected in copper slot grids coated with Formvar and subjected to poststaining with lead citrate/uranyl acetate using a Leica stainer (Leica Microsystems, Germany) according to the manufacturer’s instructions. The grid was then carbon coated for TEM imaging. Similarly, sections of 250-nm thickness were collected onto a 200 square copper mesh grids, stained, carbon coated, and used for electron tomography.

### Volume microscopy.

Volume microscopy was done with a focused ion beam scanning electron microscope (Helios 650; FEI Company, Eindhoven, The Netherlands). Milling was done with a gallium ion beam at 30 kV and 2.4 nA. The imaging was done at 1.5 keV, 800 pA, and a 6,144 × 4,096 frame size, with a 71.4-Å pixel size and a 100-Å section thickness. The images were aligned with IMOD using cross-correlation.

### Electron microscopy.

The poststained carbon coated ultrathin sections were examined using 100 KV Philips CM100 transmission electron microscope. For electron tomography and cryo-ET, we used the 2200FS cryo-transmission electron microscope (JEOL) with an omega filter. For ET, tilt series between −60° to +60° with 2° increments were recorded using SerialEM software (University of Boulder) controlling a TVIPS F416 camera (Tietz Video and Image Processing Systems). For cryo-ET, Direct Electron DE20 detector was used for imaging. Tilt series were collected at a calibrated magnification corresponding to pixel size of 3.8 Å/pix with an exposure of total dose of ∼100 e^–^/Å^2^.

### Image processing.

Electron tomograms were reconstructed using IMOD software suite (v4.9.5) (52). Patch tracking was used for aligning the tilt series collected from resin sections, and 10-nm gold fiducial markers were used for tracking and aligning the cryo-ET tilt series. Weighted back-projection was used for reconstructing all the tomograms. Manually traced segmentation of the features was carried out using “drawing” and “interpolation” in IMOD, which were further refined with UCSF Chimera (v1.12). IMOD was used for statistical measurements on 20 to 30 structures.
