# Supplementary material for: Visualizing Nudivirus Assembly and Egress
Source: mBio. 2020 Aug 11;11(4):e01333-20. doi: 10.1128/mBio.01333-20 (PMC7439470; doi:10.1128/mBio.01333-20)
Supplement: FIG S1 [file mBio.01333-20-sf001.pdf]

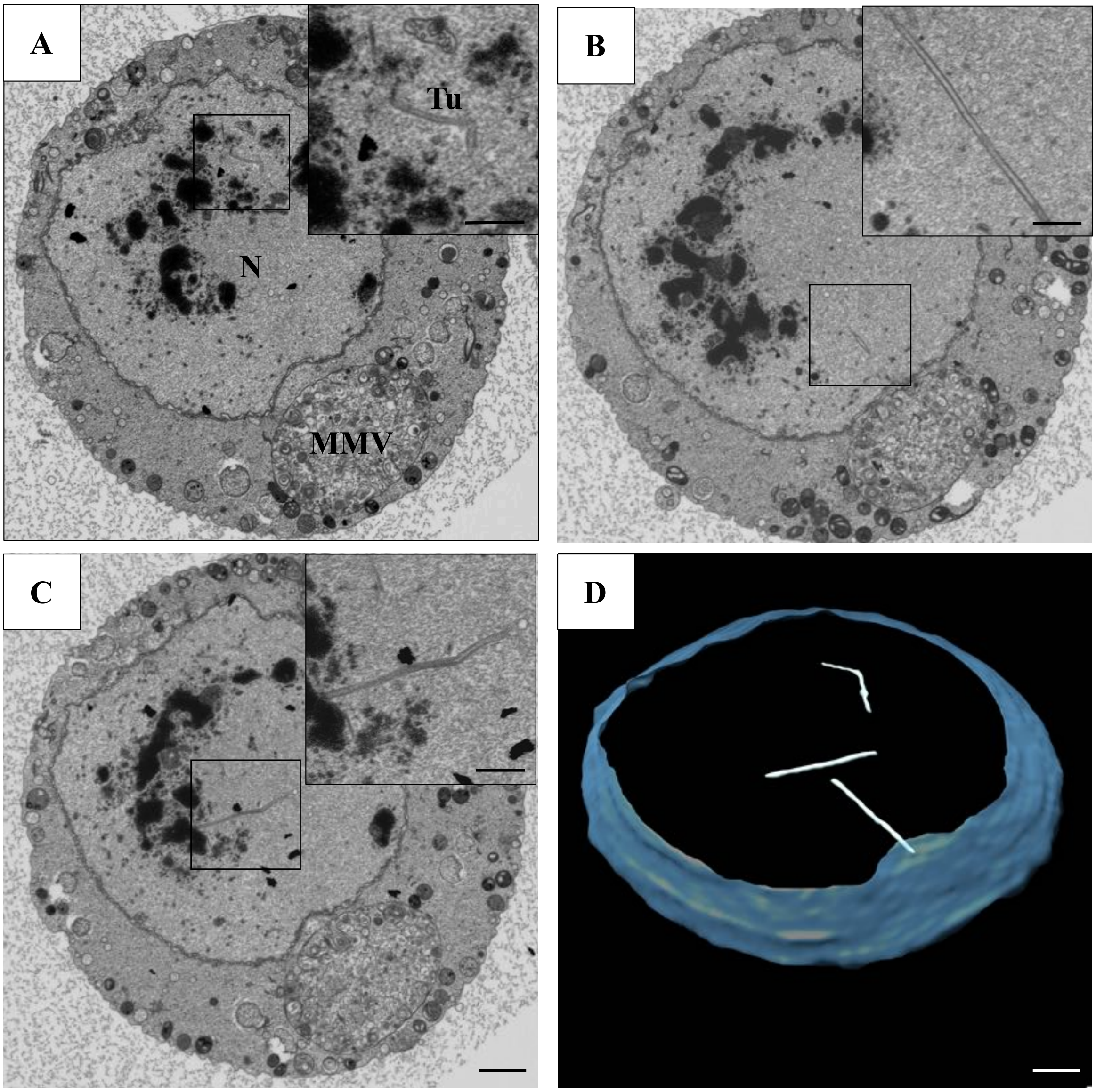

Fig S1. Volume electron microscopy displaying long tubules scattered around the entire volume of the nucleus at 72 hpi. A) Micrographs displays tubules, approximately 2-3 µm long, near the nuclear membrane (A & B) and at the center of the nucleus (C). Insets are 36 nm thick tomographic slices at higher magnification. Only part of the tubule is seen in image B, while the inset shows a full length of tubule, as the volume in the inset is rotated (by 3.6 ° and 0.1 °) along *x* and *z* axis. Scale bar of images A, B & C (and their insets) represents 1000 nm. D) Segmentation of the volume, tilted at 45 °, displays tubules scattered around the nucleus. Scale bar represents 2000 nm.
