# Supplementary material for: Visualizing Nudivirus Assembly and Egress
Source: mBio. 2020 Aug 11;11(4):e01333-20. doi: 10.1128/mBio.01333-20 (PMC7439470; doi:10.1128/mBio.01333-20)
Supplement: FIG S2 [file mBio.01333-20-sf002.pdf]

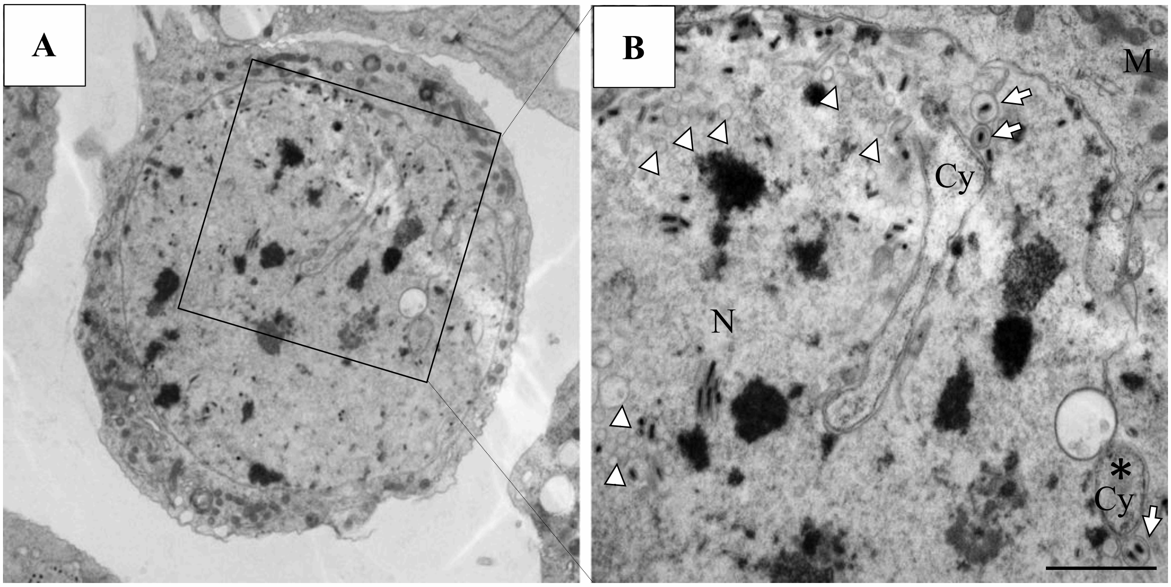

Fig S2. Invagination of the cytoplasm into the nucleus. (A) Cross-section of a cell showing cytoplasmic components invaginating into the nucleus. (B) Cytoplasmic organelles, such as ribosomes, can be clearly seen inside these invaginations at a higher magnification in the inset. Due to level of section cut, these invaginations (*) from cytoplasm (Cy), sometime encapsulating the virions (indicated by arrows), appear as double membrane vesicles. Numerous microvesicles noticed inside the nucleus are indicated arrowheads. Scale bar presents 1000 nm.
