# Supplementary material for: Visualizing Nudivirus Assembly and Egress
Source: mBio. 2020 Aug 11;11(4):e01333-20. doi: 10.1128/mBio.01333-20 (PMC7439470; doi:10.1128/mBio.01333-20)
Supplement: FIG S3 [file mBio.01333-20-sf003.pdf]

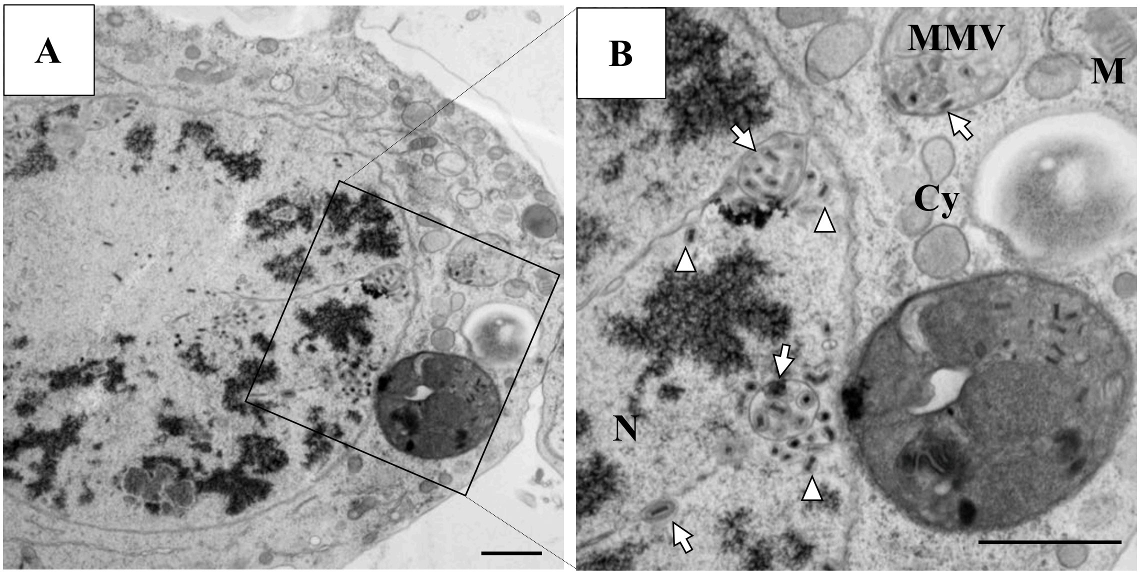

Fig S3. Distribution of virions in interchromatin space and cytoplasm. Micrograph of a cell showing virions dispersed in the nucleus and cytoplasm. The inset at higher magnification clearly shows that cytoplasmic organelles are not affected during virions replication and assembly. Virions appear scattered throughout the nucleus (N) and cytoplasm (Cy) either alone (arrowhead) or inside vesicles (arrows). Scale bar presents 1000 nm.
