# Supplementary material for: Visualizing Nudivirus Assembly and Egress
Source: mBio. 2020 Aug 11;11(4):e01333-20. doi: 10.1128/mBio.01333-20 (PMC7439470; doi:10.1128/mBio.01333-20)
Supplement: FIG S4 [file mBio.01333-20-sf004.pdf]

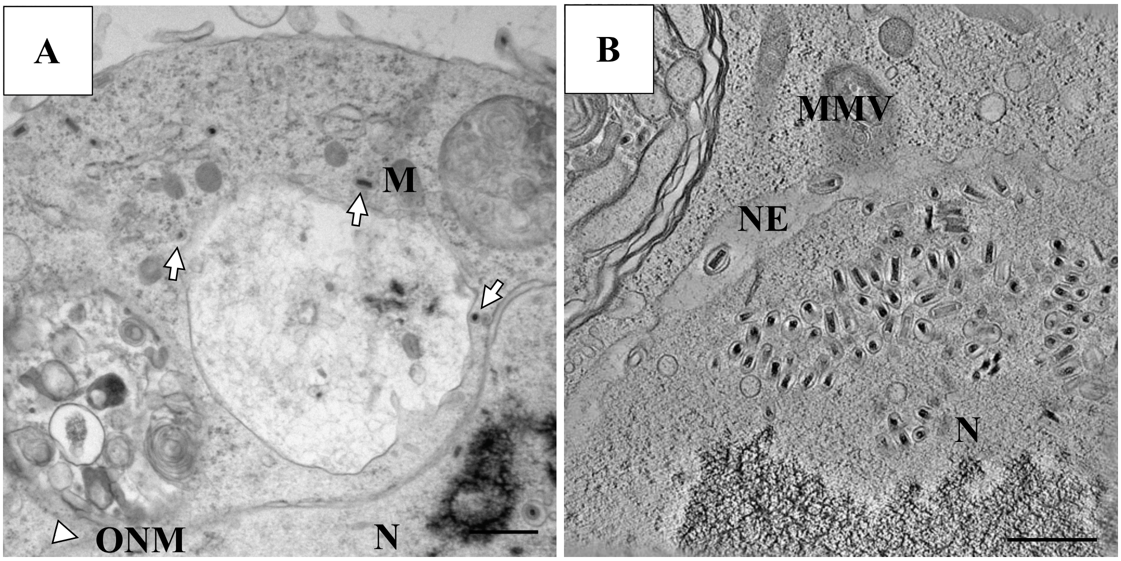


**Fig S4. Exvagination of virions into the cytoplasm.** (A) An electron micrograph displaying virions expelled into the cytoplasmic region (indicated by arrow). Association of outer nuclear membrane (ONM) with the multi membrane vesicles (MMV) is indicated with an arrowhead. (B) A 30 nm tomographic slice shows that every single virion, inside the nuclear envelope (NE), is enclosed within a vesicle, exvaginating into a MMVs. Scale bar presents 500 nm.
