# Supplementary material for: Visualizing Nudivirus Assembly and Egress
Source: mBio. 2020 Aug 11;11(4):e01333-20. doi: 10.1128/mBio.01333-20 (PMC7439470; doi:10.1128/mBio.01333-20)
Supplement: FIG S5 [file mBio.01333-20-sf005.pdf]

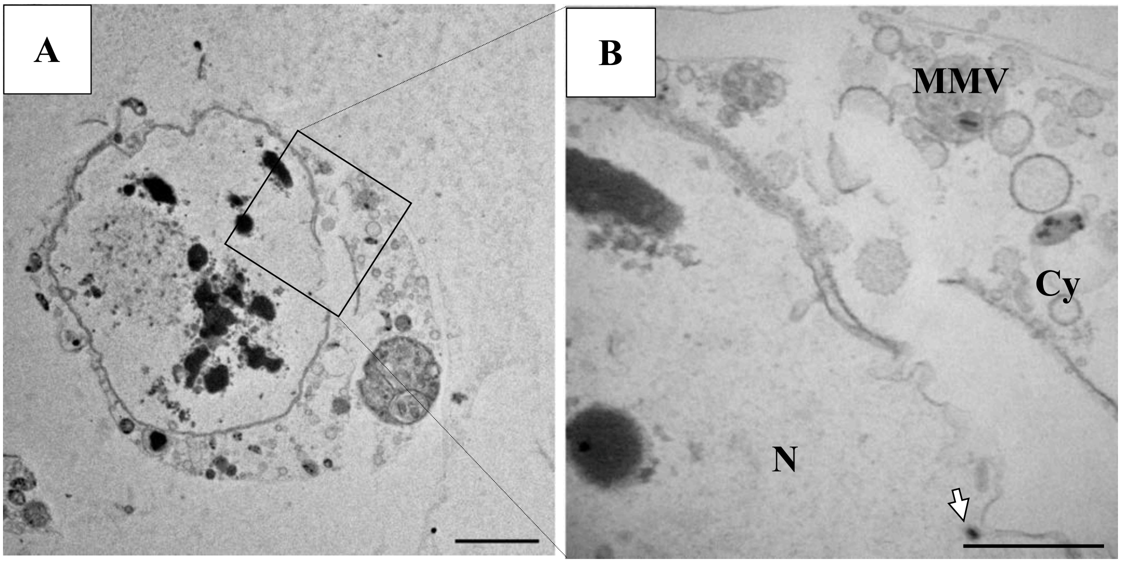

Fig S5. Cell lysis following 72 hpi with OrNV. (A) An electron micrograph of an OrNV infected cell undergoing lysis. (B) High magnification of the inset showing individual free-floating virions in the distorted nucleus (N) is indicated with an arrow. The cellular membrane is also ruptured, however, MMVs with few virions are still present. Scale bar presents 1000 nm.
